# Polygenic Risk, Trait Variables, and External Stressors in Fatal and Nonfatal Suicidal Behavior

**DOI:** 10.1001/jamanetworkopen.2025.54325

**Published:** 2026-01-15

**Authors:** Min Ji Kim, Hanga Galfalvy, Tarjinder Singh, J. John Mann

**Affiliations:** 1 Molecular Imaging and Neuropathology Division, New York State Psychiatric Institute, New York; 2 Department of Psychiatry, Columbia University Vagelos College of Physicians and Surgeons, New York, New York; 3 Department of Biostatistics, Mailman School of Public Health, Columbia University, New York, New York; 4 New York Genome Center, New York

## Abstract

**Importance:**

Suicidal behavior is heritable and influenced by various clinical traits and external stressors. Clarifying these genetic and environmental influences may guide targeted intervention.

**Objective:**

To examine the interplay among polygenic scores for suicide attempt (suicide-PGSs), clinical characteristics, and external stressors in suicidal behavior.

**Design, Setting, and Participants:**

This case-control study included genome-wide genotyping of individuals with nonfatal suicide behavior, individuals who died by suicide, and alive and deceased control individuals with no suicidal behavior or death by suicide, respectively. Data were collected in New York, New York; Montreal, Canada; and Munich, Germany, between 1991 and 2011, and analyses were performed from July 1 to October 30, 2024.

**Exposure:**

Suicide-PGSs, internal traits (hostility, impulsivity, aggression), clinical variables (depression severity, suicidal ideation, number and lethality of suicide attempts, number of depressive episodes), and external stressors (childhood abuse, recent life events).

**Main Outcomes and Measures:**

Associations between suicide-PGS and suicidal behavior, traits, clinical features, and environmental stressors were assessed via logistic regression, linear regression, and Poisson models.

**Results:**

The sample included 1699 individuals across 2 cohorts. The live cohort included 1275 participants, of whom 239 had attempted suicide (mean [SD] age, 41.8 years; 147 female [61.5%]) and 1036 were control participants who had not (mean [SD] age, 37.3 [17.3] years; 574 female [55.4%]), and the postmortem cohort included 424 individuals, of whom 294 died by suicide (mean [SD] age, 45.1 [17.0] years; 219 male [74.5%]) and 130 were control individuals who died of other causes (mean [SD] age, 49.6 [18.3] years; 102 male [78.5%]). Suicide-PGS was associated with suicide attempts in the live (odds ratio [OR], 1.35; 95% CI, 1.17-1.56) and postmortem (OR, 1.34; 95% CI, 1.07-1.70) cohorts. Among live participants who attempted suicide, higher suicide-PGS was associated with lifetime aggression severity (*b* = 0.67; 95% CI, 0.41-0.94), depression severity (*b* = 0.20; 95% CI, 0.12-0.28), and less hostility (*b* = −0.51; 95% CI, −0.82 to −0.19) but not with impulsivity or lethality. Suicide-PGS was also associated with more depressive episodes (*b* [SE], 0.11 [0.04]) but not the number of lifetime suicide attempts in the live cohort. Suicide-PGS was associated with reported childhood abuse (OR, 1.16; 95% CI, 1.02-1.33) and recent life stress (*b* [SE], 0.17 [0.05]) in the live cohort, though these stressors did not moderate genetic associations with suicide.

**Conclusions and Relevance:**

This case-control study found that genetic liability for suicide attempt was associated with clinical characteristics, internal traits, and external stressors, highlighting the complexity of genetic and environmental interactions. Larger studies with detailed phenotyping are needed to clarify genetic-environment contributions to suicide risk.

## Key Points

**Question:**

What is the association between genetic liability for suicide attempts and clinical characteristics, internal traits, and external stressors in suicidal behavior?

**Findings:**

In this case-control study of 1699 individuals, polygenic scores for suicide attempts were significantly associated with both fatal and nonfatal suicide attempts. Genetic liability for suicide attempts was associated with internal traits, including more severe lifetime aggression, more severe depression, and less overt hostility, as well as more severe external stressors, such as childhood abuse and recent life stressors, in individuals with nonfatal suicide attempts.

**Meaning:**

These findings suggest that genetic liability for suicide attempts may be mediated by both clinical and environmental factors, spanning traits associated with the diathesis for suicidal behavior; understanding the causes and prevention of suicidal behavior should consider the role of genetics.

## Teaser

This case-control study examines the association of suicidal behavior with polygenic scores for suicide attempts, clinical characteristics, and external stressors among alive and deceased cohorts from the US, Canada, and Germany.

## Introduction

Suicidal behavior is a major public health concern, accounting for approximately 700 000 deaths globally each year.^1^ Family and twin studies have indicated moderate heritability of suicidal behavior (30%-55%),^2,3,4^ attributable partly to psychiatric disorders and partly to a predisposition toward suicidal behavior that is independent of psychiatric diagnosis.^5,6^ The largest meta-analysis of genome-wide association studies (GWASs) of suicide attempts comprised 22 cohorts, including 43 871 individuals who attempted suicide, and identified 12 loci,^7^ including regions near *DRD2* and *PDE4B*, genes involved in dopaminergic signaling and linked to impulsivity and aggression. These findings suggest that genetic liability to suicide attempts may be associated with intermediate behavioral traits.

The risk for suicide attempts depends, in part, on associated major psychiatric disorders, of which the most common is major depression, as well as several internal traits, including hostility, impulsivity, and aggressiveness.^8,9,10,11,12^ Individuals with multiple suicide attempts exhibit distinct clinical characteristics compared with those with a single attempt, including more severe psychopathology with comorbid mood and psychotic disorders, personality disorders, and substance use disorders.^13,14,15,16^ Furthermore, the number, severity, and earlier onset of depressive episodes correlate with suicide attempts.^17,18^ Extending these observations, attempt lethality, which reflects planning, intent, and medical severity, captures additional clinically meaningful dimensions of suicidal behavior.^19,20^ However, the genetic underpinnings of these associations remain incompletely understood.

In addition to internal factors, external stressors, such as history of childhood abuse and recent life stressors, also influence risk for suicide attempts. Environmental stressors may interact with genetic liability in 2 ways. The first is through gene-environment correlation, in which genetic predisposition influences an individual’s likelihood of encountering adverse environments (eg, childhood abuse).^21^ The second is through gene-environment interaction, in which genetic liability moderates sensitivity to the effects of those stressors, potentially via mechanisms such as epigenetic regulation or genetics.^22,23^ Childhood abuse, in particular, may heighten stress sensitivity after puberty,^24,25,26,27,28^ while acute stressors, such as interpersonal conflict or loss often precipitate suicidal behavior.^29,30,31^

Nonfatal suicide behavior and suicide deaths represent overlapping, yet distinct phenomena. Suicide death is the most severe outcome of suicidal behavior, often occurring on the first attempt, while a prior attempt remains one of the strongest predictors of future attempts and suicide.^32,33,34,35^ Yet, biological and contextual differences suggest that these groups may differ in risk profiles.^36,37,38^ Comparing individuals with nonfatal suicide behavior with those who died by suicide is therefore essential to understanding the interplay of genetic liability, traits, and stressors across the spectrum of suicidal behaviors.

This study investigated the interplay among polygenic risk (PGS) for suicide attempt (suicide-PGS), individual trait and clinical variables associated with suicide, and external stressors (ie, childhood abuse, recent life stressors) in a well-characterized cohort representing both nonfatal suicide behavior and deaths by suicide. In this study, we examined the association between clinical variables associated with suicide and the genetic liability for suicide attempts in both live and postmortem samples, as well as how genetic liability interacts with external stressors to estimate future suicide attempts.

## Methods

### Samples

In this case-control study, samples were collected from New York, New York; Montreal, Canada; and Munich, Germany, between 1991 and 2011, and genome-wide assays were performed from 2009 through 2010. Suicidal behavior included deaths by suicide and suicide attempts. A suicide attempt was defined as a self-injurious act with at least some intent to end one’s life. Individuals who attempted suicide were identified from all 3 sites, while individuals who died by suicide were identified only from New York and Montreal. All studies were approved by local institutional review boards and ethics committees. Written informed consent was obtained from all living participants. For deceased individuals, psychological autopsy interviews were conducted after written informed consent from next of kin as approved by local institutional review boards. Deidentified postmortem samples were classified by the institutional review board of Columbia University as nonhuman participant research. All procedures complied with relevant national and institutional ethical standards and the revised Declaration of Helsinki.^39^ The study followed the Strengthening the Reporting of Observational Studies in Epidemiology (STROBE) reporting guideline.

Psychiatric diagnoses were assessed using the Structured Clinical Interview for *DSM-I *or Structured Clinical Interview for *DSM-III-R* for all case and control individuals. Live control individuals were a mixture of healthy volunteers without psychiatric diagnoses and patients with psychiatric disorders without a history of suicide attempt. Individuals who attempted suicide were primarily ascertained through clinical settings following presentation for medical care or care-seeking to enter a research study.

For postmortem samples, individuals who died by suicide were ascertained from the general population through medical examiner offices. Nonfatal suicide behavior was determined by the research team using the Columbia Classification Algorithm for Suicide Assessment,^40^ while coroners or medical examiners determined both suicide and other causes of death. Postmortem control individuals, including those who died suddenly of an accident or natural death, included a subgroup of individuals with psychiatric disorders. All case and control individuals in the postmortem group were clinically characterized using the Structured Clinical Interview for *DSM-I *through our validated psychological autopsy method.^41,42^ Further details on the demographic and clinical characteristics of the dataset have been previously published.^43^

### Measures

For participants who had attempted suicide recruited from New York and Montreal, the Beck Suicide Intent Scale was used to assess the level of intent.^44^ Suicidal ideation was measured in both case and control participants at all 3 sites using the Beck Scale for Suicidal Ideation, which consists of 19 items that evaluate suicidal thoughts and plans.^45^ Depression severity scores were obtained near the time of blood sampling in case and control participants using either the Hamilton Depression Rating Scale^46^ or the Allgemeine Depressions-Skala^47^ in all 3 sites. The *z* scores for these scales were calculated separately and then combined into 1 composite quantitative depression severity score for case participants, as described previously.^48^ Hostility was assessed using the Buss-Durkee Hostility Inventory^49^ in a subset of case and control participants in all 3 sites. In the live cohort, trait impulsivity was measured using the Barratt Impulsivity Scale Version 10,^50^ while trait aggression was evaluated using the Brown-Goodwin Aggression History^51^ score in all 3 sites. Trait aggression evaluation using the Brown-Goodwin Aggression History score was also conducted by psychological autopsy in subsets of the postmortem cohort. Lethality of the most severe suicide attempt was assessed in the live cohort using the Beck Lethality Scale (0-8, with higher scores indicating greater objective medical threat).^52^ The number of suicide attempts was counted as lifetime events, and depressive episodes were recorded as lifetime major depressive episode counts, ascertained by structured interview (live cohort) or psychological autopsy (postmortem cohort).

With regard to childhood abuse history, both live and postmortem cohorts were asked whether there was any history of physical and/or sexual abuse before age 16 years. A positive response to either physical or sexual abuse indicated yes for the history of childhood abuse; otherwise, the response was recorded as no. Recent life stress was assessed using the St Paul Ramsey Life Experience Scale,^53^ which involves questions about significant events occurring within 6 months before the blood draw for live cohort participants or before death in the postmortem cohort. The assessment covered interpersonal, occupational, and health-related events. The scale was scored by global severity (1-7, with 1 indicating none and 7 indicating catastrophic) and by mean category scores. We used the mean category scores for the analysis.

All measures were obtained for living participants through clinical interviews and self-report assessments. For decedents, comparable information was collected through structured psychological autopsy interviews with informants supplemented by review of medical and coroner records.

### Genotyping and PGS Calculation

All DNA samples were stored at −80 °C in accordance with standardized protocols to maintain sample integrity. All samples across the New York, Montreal, and Munich sites were genotyped using the HumanOmni1-Quad BeadChip array (1 014 770 single nucleotide variants; Illumina). Quality-control procedures were applied uniformly across sites.

Markers were retained if they had a minor allele frequency of 1% or more, a call rate of 95% or more, and no significant departures in control participants from Hardy-Weinberg equilibrium (*P* ≥ 1 × 10^−6^). No samples with discrepancies between reported and genetically inferred sex were detected. Genotyping call completeness less than 0.95, heterozygosity rate deviating more than 3 SDs from the mean, and duplicated individuals (identity by descent >0.95) were excluded. Ancestry was assigned via principal component analysis by projecting samples onto the 1000 Genomes reference panel. Individuals clustering within 6 SDs of the European centroid on principal components 1 and 2 were classified as European. Samples outside this range were excluded, retaining 1775 of 1823 participants (97.4% of the analytic sample), of European ancestry for analysis. The genotype data were imputed using the Michigan Imputation Server.^54^ The 1000 Genomes was chosen as the reference panel, and phasing and imputation were performed using Eagle, version 2.4^55^ and Minimac4,^56^ respectively. Among the imputed genotypes, variants whose minor allele frequency was less than 0.01 and *R*^2^ less than 0.3 were excluded.

Suicide-PGS was generated using summary statistics from the most recent and largest GWAS of suicide attempts conducted by Docherty et al.^7^ This study was based predominantly on nonfatal attempts (presence vs absence), making it relevant for estimating attempt occurrence but limited for analyzing frequency or lethality. We used the summary statistics, excluding the New York cohort samples. The PGS was computed using PRSice-2.^57^ Linkage disequilibrium clumping was applied (*r*^2 ^<0.1, 250-kb window). The PGSs were computed across *P *value thresholds (5 × 10^−8^ to >.99), and the best-fit threshold (maximizing variance explained in our sample) was selected. Final PGSs were standardized and checked for outliers before analysis.

### Statistical Analysis

Statistical analyses were performed from July 1 to October 30, 2024, using R, version 4.3.1 (R Foundation for Statistical Computing). We first evaluated the association of suicide-PGS with nonfatal suicide behavior and death by suicide using logistic regression across composite, live, and postmortem cohorts while adjusting for sex, age, and scaled principal components 1 to 5 from the principal component analysis. This approach is consistent with standard practice in PGS analyses: When sample ancestry is relatively homogeneous and outliers are removed, approximately 5 principal component are typically sufficient to correct for residual population stratification.^58,59^

We also conducted a sensitivity analysis restricting control participants to those with major depressive disorder (MDD) but no suicide attempt (n = 264), given that nearly all live participants who attempted suicide had MDD (237 of 239 [99.2%]). We reestimated the association between suicide-PGSs and attempt status using logistic regression adjusted for the same covariates as in the primary model to assess whether the observed associations reflected suicide-specific liability vs genetic liability shared with MDD.

Next, separate linear regressions were conducted with the PGS as the estimator and the severity scores for (1) suicide intent, (2) suicidal ideation, (3) depression, (4) hostility, (5) impulsivity, and (6) aggression as response variables. Sex, age, and the first 5 principal components were included as covariates. Nagelkerke *R*^2^ value was used to estimate the proportion of variance in each clinical measure explained by each PGS. The significance level for each regression analysis was set at .008, accounting for the analysis of suicide-PGS for 6 state and trait variables (Bonferroni correction cutoff of .05 divided by 6).

The number of lifetime suicide attempts and the number of depressive episodes were analyzed using Poisson models. To mitigate zero inflation from control participants, analyses were restricted to relevant subgroups: The number of attempts was modeled only among participants who attempted suicide, and depressive episodes were modeled only among participants with 1 or more depressive episodes who had attempted suicide. All models were adjusted for age, sex, and the first 5 principal components.

Gene-environment correlations were evaluated by examining the association between suicide-PGS and environmental measures. For the binary childhood abuse response variable, a logistic regression model was used that controlled for sex, age, and the first 5 principal components. Similarly, linear regression models were used with the same covariate adjustments for continuous measures of recent life stress. Gene-environment interactions were assessed using logistic regression models with suicide attempt and death by suicide as the binary outcome. The models included the main effects for the suicide-PGS and the environmental variables, as well as interaction terms between them.

For all model-based analyses, we used complete-case analysis, restricting each model to participants with nonmissing values on the outcome and all included covariates. All statistical tests were 2-sided, with *P* < .05 considered statistically significant unless otherwise specified.

## Results

### Sample Characteristics

The final analytic sample included 1699 individuals across 2 cohorts. The live cohort included 1275 participants, of whom 239 had attempted suicide (case participants) (mean [SD] age, 41.8 [12.0] years; 147 female [61.5%] and 92 male [38.5%]) and 1036 had no history of suicide attempt (control participants) (mean [SD] age, 37.3 [17.3] years; 574 female [55.4%] and 462 male [44.6%]). The postmortem cohort included 424 individuals, of whom 294 died by suicide (mean [SD] age, 45.1 [17.0] years; 75 female [25.5%] and 219 male [74.5%]) and 130 died of other causes (mean [SD] age, 49.6 [18.3] years; 28 female [21.5%] and 102 male [78.5%]). Sample characteristics are detailed in Table 1. In the live cohort, MDD was more prevalent in case participants compared with control participants (237 [99.2%] vs 264 [25.5%]), and in the postmortem cohort, MDD was more prevalent among individuals who died by suicide compared with those who died of other causes (108 [36.7%] vs 13 [10%]). In the live cohort, case participants had significantly higher scores than control participants for suicidal ideation (mean [SD], 8.3 [8.4] vs 3.7 [6.1]; *P* < .001), trait aggression (mean [SD], 14.0 [5.1] vs 12.0 [3.6]; *P* < .001), impulsivity (mean [SD], 43.7 [7.5] vs 41.5 [6.8]; *P* < .001), and *z*-scored depressive symptoms (mean [SD], 0.2 [0.9] vs −1.07 [1.1]; *P* < .001).

**Table 1.** Sample Characteristics in the Live and Postmortem Cohorts

Characteristic	Live cohort		*P* value	Postmortem cohort		*P* value
	Control participants (n = 1036)	Partipants who attempted suicide (n = 239)		Control decedents (n = 130)	Individuals who died by suicide (n = 294)	
**Demographic variables**						
Age, mean (SD), y	37.3 (17.3)	41.8 (12.0)	<.001	49.6 (18.3)	45.1 (17.0)	.01
Sex, No. (%)						
Female	574 (55.4)	147 (61.5)	.10	28 (21.5)	75 (25.5)	.37
Male	462 (44.6)	92 (38.5)		102 (78.5)	219 (74.5)	
**Comorbid psychiatric disorder, No. (%)**						
MDD	264 (25.5)	237 (99.2)	<.001	13 (10.0)	108 (36.7)	<.001
Bipolar disorder	0	0		3 (2.3)	16 (5.4)	
Psychotic disorder	1 (0.1)	2 (0.8)		4 (3.1)	25 (8.5)	
No diagnosis	771 (74.3)	0		74 (67.3)	17 (8.1)	
Unknown diagnosis	0	0		20 (15.4)	83 (28.2)	
**Suicide-associated variables, mean (SD) [denominator]** ^a^						
Suicide intent	0	14.8 (5.6) [177]		NA	ND	<.001
Suicidal ideation	3.7 (6.1) [246]	8.3 (8.4) [122]	<.001	NA	ND	
Trait aggression	12.0 (3.6) [581]	14.0 (5.1) [211]	<.001	10.6 (3.9) [96]	13.7 (5.5) [166]	
Hostility	11.3 (5.0) [489]	10.3 (4.1) [164]	.017	ND	ND	
Impulsivity	41.5 (6.8) [569]	43.7 (7.5) [166]	<.001	ND	ND	
Depression severity score	−1.1 (1.1) [526]	0.2 (0.9) [179]	<.001	ND	ND	
Lethality	0	2.7 (2.0) [149]		2.0 (6.0) [5]	NA [91]	
Count						
No. of suicide attempts	0	2.0 (1.6) [239]		0.03 (0.18) [123]	1.3 (0.8) [267]	<.001
No. of depressive episodes	1.2 (3.4) [575]	3.6 (5.2) [201]	.001	0.2 (0.5) [73]	1.2 (1.0) [109]	<.001
**Environmental variables**						
Childhood abuse, No./total No. (%)^a^	233/1032 (22.6)	87/230 (37.8)	<.001	3/106 (2.8)	24/253 (9.5)	.048
Stress, mean (SD) [denominator]^a^	0.8 (1.2) [410]	1.6 (1.6) [121]	<.001	1.7 (0.7) [73]	2.2 (0.7) [95]	<.001

Abbreviations: MDD, major depressive disorder; NA, not applicable for the given group or measure; ND, missing or unavailable data.

^a^ Denominators vary by row due to variable-specific missingness.

The mean (SD) number of lifetime suicide attempts was 2.0 (1.6) in the live cohort and 1.3 (0.8) in the postmortem cohort (Table 1). The mean (SD) number of depressive episodes was significantly higher among individuals who attempted suicide in both the live (3.6 [5.2] vs 1.2 [3.4]; *P* = .001) and postmortem (1.2 [1.0] vs 0.2 [0.5]; *P* < .001) cohorts. Childhood abuse was significantly more common in live cohort case participants compared with control participants (87 [37.8%] vs 233 [22.6%]; *P* < .001) and in individuals who died by suicide compared with those who died of other causes (24 [9.5%] vs 3 [2.8%]; *P* = .048) in the postmortem cohort. Similarly, recent life stress scores were significantly higher in the live cohort between case and control participants (mean [SD], 1.64 [1.62] vs 0.78 [1.20]; *P* < .001) and in postmortem group between individuals who died by suicide compared with those who died of other causes (mean [SD], 2.23 [0.69] vs 1.72 [0.73]; *P* < .001).

### Association Between Suicide Clinical Severity Scores and Suicide-PGSs

**Figure 1.**
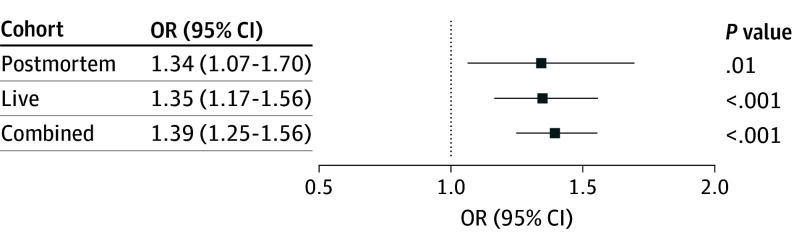
Suicide Polygenic Score–Estimated Suicide Attempts in the Postmortem, Live, and Combined Cohorts OR indicates odds ratio.

Suicide-PGSs^7^ were used to estimate suicidal behavior across the postmortem, live, and combined cohorts (Figure 1). In the postmortem cohort, the suicide-PGS estimated suicide death (odds ratio [OR], 1.34; 95% CI, 1.07-1.70; *P* = .01). Similarly, in the live cohort, the suicide-PGS showed a comparable odds for estimating suicide attempt (OR, 1.35; 95% CI, 1.17-1.56; *P* < .001). In the combined cohort, the suicide-PGS also estimated suicide attempt or death by suicide (OR, 1.39; 95% CI, 1.25-1.56; *P* < .001). In a live cohort–only sensitivity analysis, we compared case participants with control participants with MDD (n = 264). The suicide-PGS was not significantly different between groups (OR, 1.12; 95% CI, 0.93-1.35; *P* = .25), suggesting that previous associations may partly reflect liability shared with MDD.

**Figure 2.**
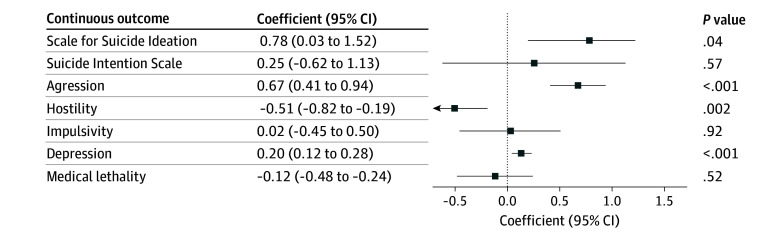
Suicide Polygenic Score–Estimated Suicide-Associated Variables

We then examined the ability of suicide-PGSs to estimate clinical and individual traits known to be associated with suicide attempt in the live cohort (Figure 2). The suicide-PGS showed a significant positive association with the severity of suicidal ideation in the live cohort (*b* = 0.78; 95% CI, 0.03-1.52; *P* = .04); however, this did not remain significant after applying the Bonferroni correction cutoff of *P* = .05/6 = .008. Hostility scores were negatively associated with suicide-PGS (*b* = −0.51; 95% CI, −0.82 to −0.19; *P* = .002), whereas depression scores (*b* = 0.20; 95% CI, 0.12-0.28; *P* < .001) and lifetime aggression severity scores (*b* = 0.67; 95% CI, 0.41-0.94; *P* < .001) were positively associated in the live cohort. No significant associations were observed between suicide-PGS and suicide intent or impulsivity scores in the live cohort. Suicide-PGS was not associated with medical lethality of the suicide attempt in the live cohort.

The suicide-PGS score and number of lifetime suicide attempts among individuals who attempted suicide revealed no significant association across combined, live, and postmortem cohorts (Table 2). In the model, female sex was associated with an increased number of suicide attempts in the combined cohort (*b* [SE], 0.26 [0.07]; *P* < .001). Among individuals with MDD who attempted suicide, suicide-PGS was associated with the number of depressive episodes in the live cohort (*b* [SE], 0.11 [0.04]; *P* = .009) (Table 2). Female sex was associated with the number of depressive episodes in the combined cohort (*b* [SE], 0.21 [0.07]; *P* = .004) but not in the separate live or postmortem cohorts.

**Table 2.** Suicide-PGS as an Estimator of the Number of Lifetime Suicide Attempts and Depressive Episodes Among Individuals Who Attempted Suicide^a^

Group	Suicide-PGS	Age	Sex
**No. of suicide attempts among individuals who attempted suicide**			
Combined (n = 506)			
*b* (SE)	0.02 (0.51)	−0.002 (0.002)	0.26 (0.07)
RR (95% CI)	1.02 (0.95-1.10)	1.00 (0.994-1.002)	1.29 (1.13-1.48)
*P* value	.51	.32	<.001
Live (n = 239)			
*b* (SE)	0.07 (0.05)	−0.002 (0.004)	0.17 (0.10)
RR (95% CI)	1.07 (0.97-1.17)	1.00 (0.990-1.006)	1.19 (0.98-1.44)
*P* value	.17	.63	.08
Postmortem (n = 267)			
*b* (SE)	−0.03 (0.06)	−0.001 (0.003)	0.04 (0.13)
RR (95% CI)	0.97 (0.87-1.09)	1.00 (0.993-1.005)	1.04 (0.81-1.33)
*P* value	.64	.78	.77
**No. of depressive episodes among individuals with >1 depressive episode who attempted suicide **			
Combined (n = 286)			
*b* (SE)	0.06 (0.04)	−0.004 (0.003)	0.21 (0.07)
RR (95% CI)	1.06 (0.99-1.14)	1.00 (0.990-1.002)	1.23 (1.07-1.41)
*P* value	.11	.12	.004
Live (n = 201)			
*b* (SE)	0.11 (0.04)	−0.001 (0.003)	0.02 (0.08)
RR (95% CI)	1.12 (1.03-1.21)	1.00 (0.993-1.005)	1.02 (0.87-1.19)
*P* value	.009	.82	.82
Postmortem (n = 85)			
*b* (SE)	−0.14 (0.11)	−0.002 (0.005)	0.03 (0.20)
RR (95% CI)	0.87 (0.70-1.09)	1.00 (0.988-1.008)	1.03 (0.69-1.52)
*P* value	.22	.74	.90

Abbreviations: RR, rate ratio; suicide-PGS, polygenic score for suicide attempt.

^a^ All models were adjusted for age, sex, and the first 5 principal components.

### Gene-Exposure Correlation and Interactions

Table 3 presents the gene-exposure correlation for suicide-PGS associated with reported abuse and recent life stress across the combined, live, and postmortem cohorts. There was a positive association between reported childhood abuse and suicide-PGS in the live cohort (OR, 1.16; 95% CI, 1.02-1.33; *P* = .02), whereas in the postmortem cohort, there was no association (OR, 1.07; 95% CI, 0.70-1.67; *P* = .75), perhaps due to a smaller sample size and lower reported rates of abuse. Suicide-PGS was associated with recent life stress in both the combined (*b* [SE], 0.19 [0.05]; *P* < .001) and live (*b* [SE], 0.17 [0.05]; *P* = .001) cohorts, suggesting that individuals with higher genetic risk for suicide may be more likely to report recent life stressors. Gene-environment interaction analyses with suicide attempt and death as a response variable did not yield statistically significant results with either childhood abuse or recent life stress under multiplicative models.

**Table 3.** Gene-Exposure Correlations and Interactions Between Suicide-PGS and External Factors^a^

Variable	Combined cohort, OR (95% CI)	*P* value	Live cohort, OR (95% CI)	*P* value	Postmortem cohort, OR (95% CI)	*P* value
**Gene-exposure correlation**						
Lifetime abuse with PGS	1.12 (0.99-1.27)	.07	1.16 (1.02-1.33)	.02	1.07 (0.70-1.67)	.75
Recent life stress with PGS, *b* (SE)	0.19 (0.05)	<.001	0.17 (0.05)	.001	0.11 (0.03)	.08
**Gene-exposure interactions (multiplicative)**						
PGS × lifetime abuse	0.95 (0.73-1.24)	.72	1.01 (0.74-1.37)	.95	0.65 (0.10-2.45)	.57
PGS × recent life stress	0.92 (0.80-1.06)	.25	0.95 (0.81-1.12)	.54	0.76 (0.42-1.43)	.38

Abbreviations: OR, odds ratio; PGS, polygenic score; suicide-PGS, polygenic score for suicide attempt.

^a^ All models were adjusted for age, sex, and the first 5 principal components.

## Discussion

This case-control study investigated the interplay of suicide-PGS, clinical traits, and stressors in fatal and nonfatal suicide behavior. We found that suicide-PGS was associated not only with fatal and nonfatal suicide behavior, but also with key internal traits (depression severity, hostility, aggression) and external stressors (childhood abuse, recent life stress), supporting a complex gene-environment model.

According to the suicide stress-diathesis model, suicidal behavior arises from the interplay of stress and diathesis.^5^ Diathesis involves trait factors, including mood regulation, impulsivity, aggression, and cognitive distortions.^5,60^ Although symptoms such as suicidal ideation or depression fluctuate, suicide-PGS may index a stable, trait-like liability (diathesis) that includes susceptibility to worsening symptoms under stress. Constructs such as impulsivity and hostility are also trait-like and estimative of suicidal behavior, supporting our correlational analyses.^8,61,62,63,64^

Our study found quantitative associations between suicide-PGS and the severity of lifetime aggression, hostility, impulsivity, depression, and suicidal behaviors. Aggression and impulsivity are established risk factors for suicide clinically and biologically.^65,66,67,68^ However, impulsivity alone may not translate directly into lethal suicide behavior, as planned suicides typically involve less impulsivity but higher intent and lethality,^69,70,71^ aligning with our findings that show a positive association between suicide-PGS and aggression severity but not impulsivity.

The higher suicide-PGS correlated with more suicidal behavior, aggression, severe depression, and depressive episodes but inversely with hostility, which is an emotion without a behavior. This finding suggests that the suicide-PGS may be associated with the transition of emotion (depression, hostility) into behavior or action (suicide attempt, aggression). We did not find an association of the suicide-PGS with suicidal ideation severity. In the live cohort, hostility scores were not elevated in case participants compared with control participants, and the suicide-PGS showed a significant negative association with hostility. This finding further suggests that polygenic liability for suicide may be more strongly tied to behavioral expression (eg, aggression) than to attitudinal traits, such as hostility. Hostility, as assessed by the Buss-Durkee Hostility Inventory, is multidimensional (assault, irritability, resentment, guilt, etc).^49^ Its association with suicide may be driven by specific cognitive rather than behavioral components,^10^ and subscales that overlap with depressive symptoms (eg, guilt, resentment) may contribute to mixed findings. These results imply that genetic liability may index pathways that translate distress into action, consistent with the stress-diathesis model.

Individuals who attempt suicide more than once exhibit more severe psychopathology than those who attempt only once.^13,14,15^ The suicide-PGS estimated the occurrence but not the frequency of suicide attempts, suggesting that this PGS estimates the likelihood rather than the recurrence of attempts. We also found no association with the medical lethality of attempts in the live cohort. This pattern may have originated from the discovery GWAS design from which the suicide-PGS was derived, which primarily contrasted the presence vs the absence of suicide attempt and did not model lethality or recurrence.^7^ Thus, the polygenic signal may be enriched for risk pathways facilitating the transition from ideation to action but not factors that differentiate medical danger.

We found novel associations between suicide-PGS and external stressors. Gene-exposure correlation suggests that external stressors are partly influenced by genetic factors.^21^ Twin studies have reported heritability estimates for stressful life events, ranging from 20% to 50%.^72,73^ Evocative or reactive gene-environment correlation explains the association between genetically influenced behavior (or symptoms) and the reactions of other people to this behavior.^21,28,74^ Our study found that suicide-PGS correlated with increased reports of childhood abuse and recent stressors, suggesting that genetically predisposed individuals may experience more adversity and stress due to genetic factors, contributing to elevated suicide risk via combined genetic and environmental effects.

### Limitations

This study had several limitations. First, the postmortem case cohort was smaller than both the live and postmortem control cohorts. Second, the suicide-PGS association was comparable for nonfatal suicide behavior and death by suicide. While some studies have suggested that fatal and nonfatal suicide behaviors are distinct,^4,75,76^ our PGS findings support the possibility that they lie on a severity spectrum. We conducted separate analyses of the combined, live, and postmortem cohorts, but results were mostly comparable. The GWASs for suicide death are limited. Docherty et al^7^ analyzed a cohort of 35 786 individuals that included only 4692 (13%) who died by suicide. Third, clinical ascertainment found high MDD prevalence (99.2%) in live cohort participants who attempted suicide. The PGS association was not significant when comparing individuals with MDD and who attempted suicide with control individuals with MDD only, suggesting that the signal may be partly shared with MDD liability rather than purely suicide specific. Future studies should include cohorts with and without MDD. Finally, while we controlled for sex, we could not rule out selection bias or fully explore the known epidemiologic sex imbalances in suicide,^77^ which may stem from unmeasured factors such as diagnostic bias or differences in care-seeking.

## Conclusions

This case-control study is, to our knowledge, the most extensive investigation to date of the interplay among polygenic risk for suicide attempt, individual trait and state variables, and external stressors in both live and postmortem cohorts. The suicide-PGS was associated with hostility, depression, and aggression and estimated the number of depressive episodes. In addition, there was an association between suicide-PGS and both reported childhood abuse and recent life stressors, suggesting an indirect genetic path to suicidal behavior via moderating external life factors. Larger longitudinal studies are needed to clarify these associations.

## Notes

Supplement.
Data Sharing Statement
