# Supplementary material for: Polygenic Risk, Trait Variables, and External Stressors in Fatal and Nonfatal Suicidal Behavior
Source: JAMA Netw Open. 2026 Jan 15;9(1):e2554325. doi: 10.1001/jamanetworkopen.2025.54325 (PMC12809366; doi:10.1001/jamanetworkopen.2025.54325)
Supplement: Supplement. — Data Sharing Statement [file jamanetwopen-e2554325-s001.pdf]

**Data Sharing Statement**

**Data**
**Data available:** No

**Additional Information**
**Explanation for why data not available:** Due to the sensitive nature of the data and the need to protect participant confidentiality, the data will not be shared.
